# Linear CT-scan measurements of cerebral ventricles in senile Poodle dogs

**DOI:** 10.29374/2527-2179.bjvm004922

**Published:** 2023-05-30

**Authors:** Glauce Vaz Diniz Araújo, Paulo de Souza-Junior, Shirley Viana-Peçanha, Carlos Augusto dos Santos-Sousa, Marcia Torres Ramos, Fernanda Coelho Simas Bernardes, Marcelo Abidu-Figueiredo

**Affiliations:** 1 Veterinarian, Departamento de Anatomia Animal e Humana, Instituto de Ciências Biológicas e da Saúde (ICBS), Universidade Federal Rural do Rio de Janeiro (UFRRJ). Seropédica, Seropédica, RJ, Brazil.; 2 Veterinarian, DSc., Laboratório de Anatomia Animal, Universidade Federal do Pampa (UNIPAMPA). Uruguaiana, Uruguaiana, RS, Brazil.; 3 Veterinarian, Programa de Pós-graduação em Medicina Veterinária da Universidade Federal Fluminense (UFF), Niterói, RJ, Brazil.; 4 Veterinarian, DSc., Centro de Ciências Biológicas e da Natureza, Universidade Federal do Acre (UFAC), Rio Branco, AC, Brazil.; 5 Veterinarian, Curso de Medicina Veterinária, Universidade Estácio de Sá (UNESA), Niterói, RJ, Brazil; 6 Veterinarian, Programa de Pós-graduação em Ciência Animal da Universidade Estadual de Santa Cruz (UESC), Ilhéus, BA, Brazil.; 7 Veterinarian, DSc., Departamento de Anatomia Animal e Humana, ICBS, UFRRJ. Seropédica, Seropédica, RJ, Brazil.

**Keywords:** computed tomography, diagnostic imaging, lateral ventricles, third ventricle, tomografia computadorizada, diagnóstico por imagem, ventrículos laterais, terceiro ventrículo

## Abstract

Breed traits seem to influence the dimensions of the cerebral ventricles in dogs. The ratios between the ventricles and the brain are crucial diagnostic criteria for suspected canine cognitive dysfunction (CCD). This study aimed to establish linear computed tomography (CT)-scan measurements of the cerebral ventricles in 55 Poodle dogs aged >7 years. To this end, cross-sectional CT images were evaluated. The measurements in the whole sample were: height of the right ventricle, 6.0 ± 1.6 mm; height of the left ventricle, 5.8 ± 1.6 mm; width of the right ventricle, 6.9 ± 1.4 mm; width of the left ventricle, 7.0 ± 1.3 mm; height of the third ventricle, 3.4 ± 0.8 mm; height of the right cerebral hemisphere, 39.5 ± 2.0 mm; and height of the left cerebral hemisphere, 40.2 ± 2.6 mm. The average ventricular measurements were higher in dogs older than 11 years (p < 0.05). However, the average ratio of the ventricle height to the height of the brain did not reveal differences between age groups, sex, or antimeres. In addition, none of the images showed fused lateral ventricles. Thus, these data can assist in interpreting ventricle size in senile Poodle dogs (aged >7 years).

## Resumo

Características raciais parecem influenciar as dimensões dos ventrículos cerebrais em cães. As proporções entre os ventrículos e o cérebro são cruciais para os casos suspeitos de disfunção cognitiva canina (DCC). Este estudo objetivou estabelecer medidas lineares dos ventrículos cerebrais de 55 cães da raça Poodle com idade acima de sete anos. Para tal, foram avaliadas imagens de cortes transversais obtidas por tomografia computadorizada. As medidas na amostragem total foram: altura do ventrículo direito 6,0 ± 1,6 mm, altura do ventrículo esquerdo 5,8 ± 1,6 mm, largura do ventrículo direito 6,9 ± 1,4 mm, largura do ventrículo esquerdo 7.0 ± 1,3 mm, altura do terceiro ventrículo 3,4 ± 0,8 mm, altura do hemisfério cerebral direito 39,5 ± 2,0 mm e altura do hemisfério cerebral esquerdo 40,2 ± 2,6 mm. As médias das medidas ventriculares foram maiores em cães acima de 11 anos de idade (p < 0,05). Contudo, a relação entre altura ventricular e altura cerebral não revelou diferenças entre grupos etários, sexo ou antímeros. Além disso, nenhuma imagem revelou ventrículos fusionados. Assim, os dados do presente estudo podem auxiliar na interpretação das dimensões ventriculares em cães da raça Poodle acima dos sete anos de idade.

## Introduction

There is diagnostic interest in the ratios between the ventricles and the brain in dogs, especially in cases with suspected canine cognitive dysfunction (CCD). This disease typically affects senile dogs (≥8 years) and resembles human Alzheimer's disease (Dewey et al., 2019; Fast et al., 2013; Schütt et al., 2015). Macroscopically, CCD presents with structural abnormalities such as cerebral atrophy, enlargement of the ventricles, and thickening of the meninges (Dewey et al., 2019; Schütt et al., 2016). A combination of a thorough patient history, owner-reported behaviors, clinical cognitive tests, and assessment of ventricle size using imaging examinations may support the diagnosis of CCD (Pilegaard et al., 2017).

Although magnetic resonance imaging (MRI) is the best method for visualizing structural changes in the brain, many diagnostic centers have no devices available for animals, and examination costs are prohibitive for many pet owners. Thus, computed tomography (CT) scans may represent an accessible alternative for evaluating the shape, symmetry, and size of the lateral and third ventricles (Doiche et al., 2022). In a comparative study of methods for measuring ventricle sizes, linear measurements were efficient in detecting simple dilations. Furthermore, the ventricular height is positively correlated with the ventricular area and volume (Woo et al., 2010).

Despite the availability of CT scans, standardized measurements of brain structures and their ventricular systems in different skull morphotypes and breeds need to be made available (Doiche et al., 2022; Kii et al., 1997). In addition, previous studies using CT and MRI demonstrated that the adoption of a single pattern of ventricular measurements cannot be applied to all domestic dogs because of the wide variety of skull sizes and shapes and, consequently, that of the brain and its related structures (Doiche et al., 2022; Esteve-Ratsch et al., 2001; Schröder et al., 2006; Vite et al., 1997).

For linear analysis of the ventricular system using CT scans, the heights of the lateral and third ventricles are routinely measured with the "distance" tool by drawing a straight line parallel to the longitudinal fissure at the interthalamic adhesion. These measurements are broadly applied, regardless of the wide variation in the canine skull shapes and sizes (Doiche et al., 2022; Esteve-Ratsch et al., 2001; Schröder et al., 2006; Vite et al., 1997; Woo et al., 2010).

Poodles are the most common breed of dogs in many countries (Federation Cynologique Internationale, 2013; Yamato et al., 2006). Thus, this study aimed to present the average linear CT-scan measurements and indices from the cerebral ventricles in senile Poodles.

## Material and methods

This study analyzed cross-sectional images of 55 Poodle dogs aged ≥7 years at the level of interthalamic adhesions. Animals that presented structural brain lesions or gross asymmetries in CT images were excluded. This study consisted of a retrospective analysis of images and clinical histories in a database; therefore, it was exempted from approval by the Ethics Committee on the Use of Animals.

Patient images were divided into four groups: **M1**, male dogs aged 7–10 years; **M2**, male dogs aged ≥11 years; **F1**, female dogs aged 7–10 years; and **F2**, female dogs aged ≥11 years. The scans were done in a Siemens^®^ Somatom AR Star CT scanner from a veterinary diagnostic center. The cross-sections were acquired with 2 mm thickness and 2 mm increments between them, using an 83 mA/110 kVp technique. The dogs were always positioned in ventral/sternal recumbency with the hard palate parallel to the gantry table and the thoracic limbs positioned caudally to the scanning field. A scout radiograph was obtained, and the cross-sections were programmed from the foramen magnum level to the rostral limit of the nasal cavity. In addition, a “CT brain” filter (window level 35 and width 150) was used to visualize nervous tissue.

**Figure 1:**
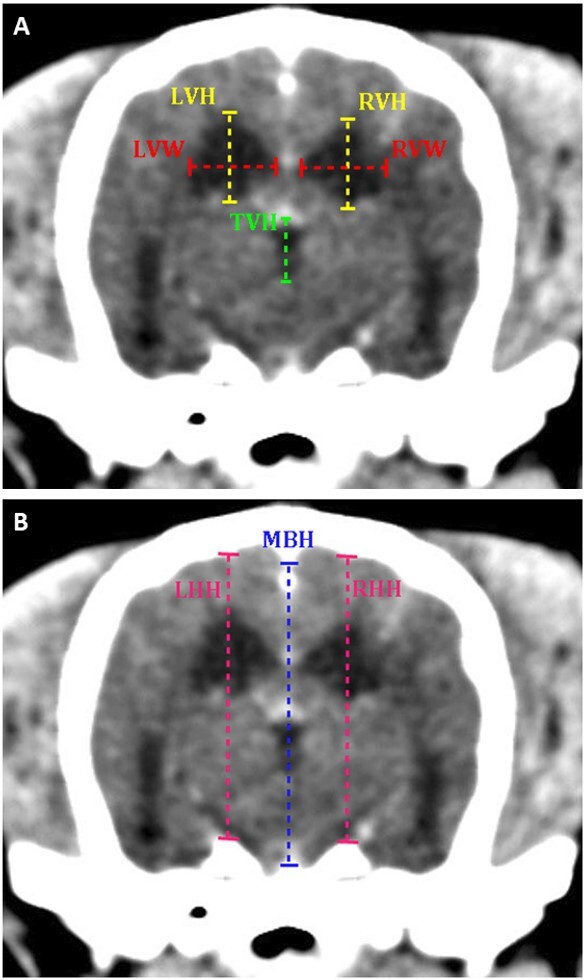
CT scan cross-section at the level of interthalamic adhesion of a 16-year-old female Poodle dog. (A) RVH, height of the right ventricle; LVH, height of the left ventricle; RVW, width of the right ventricle; LVW, width of the left ventricle; TVH, height of the third ventricle; (B) RHH, height of the right hemisphere; LHH, height of the left hemisphere; MBH, height of the brain at the midline.

The measurements were performed using the “distance” tool of the MedWork^®^ software (Table 1 and Figure 1). The ratios between the heights of the ventricles and cerebral hemispheres and between the heights of the third ventricle and the medial encephalon (RVH: RHH, LVH: LHH, and TVH: MBH) were calculated and expressed as percentages.

**Table 1:** List of measurements in cross-sectional images at the level of interthalamic adhesion of Poodle dogs ≥7 years.

**Measurement**	**Description**
**RVH** and **LVH**	Height of the right (RVH) or left (LVH) ventricle measured from the most ventral to the most dorsal point of the ventricle wall
**RVW** and **LVW**	Width of the right (RVW) or left (LVW) ventricle measured from the most lateral to the most medial point of the ventricle wall
**TVH**	Height of the third ventricle measured from the most ventral to the most dorsal point of the ventricle wall
**RHH** and **LHH**	Height of the right (RHH) or left (LHH) cerebral hemisphere at the level of the ventricle
**MBH**	Height of the encephalon at the level of the third ventricle (median fissure)

The averages and standard deviations of the lateral ventricle, third ventricle, and encephalon measurements were calculated. The Kolmogorov–Smirnov (KS) test was used to evaluate the normality of the distribution of each variable. An unpaired Student's t-test was used to compare measurements between antimeres from the same group and between sexes and ages. One-way ANOVA (complemented by Tukey's test) was used to compare the ratios between the four groups. In both tests, statistical significance was set at p < 0.05. Statistical analyses were performed using GraphPad Prism 5 software.

## Results

The number of animals evaluated and the average age of the dogs in each category are shown in Table 2. A total of 55 dogs were included in the study. Considering the entire sample (n = 55), the average measurements of the cerebral ventricles and cerebral hemispheres were: RVH 6.0 ± 1.6 mm; LVH 5.8 ± 1.6 mm; RVW 6.9 ± 1.4 mm; LVW 7.0 ± 1.3 mm; TVH 3.4 ± 0.8 mm; RHH 39.5 ± 2.0 mm; and LHH 40.2 ± 2.6 mm. The measurement values by group are listed in Table 3. No differences were observed between the left and right lateral ventricles or the heights of the left and right hemispheres (p > 0.05).

**Table 2:** Group division according to age and sex and the average age in each group

**Group**	**Age/Sex**	***n***	***Average age***
**M1**	7–10-year-old male dogs	9	8.4 y/o
**M2**	≥11-year-old male dogs	17	13.5 y/o
**F1**	7–10-year-old female dogs	6	7.5 y/o
**F2**	≥11-year-old female dogs	23	12.9 y/o
**Total**		55	11.8 y/o

y/o, years old.

**Table 3:** Measurements from cross-sectional CT images in Poodle dogs, separated by groups

**Groups**	**Average ± standard deviation (mm)**		***p*-value**
	**RVH**	**LVH**	
**M1**	5.2 ± 0.7	4.7 ± 1.3	0.39
**M2**	6.1 ± 1.5	6.1 ± 1.3	0.98
**F1**	6.2 ± 1.8	5.6 ± 2.0	0.46
**F2**	6.2 ± 1.7	6.0 ± 1.8	0.78
	**RVW**	**LVW**	
**M1**	6.2 ± 1.6	6.1 ± 1.4	0.86
**M2**	7.0 ± 1.3	7.4 ± 1.0	0.36
**F1**	6.5 ± 1.5	6.9 ± 1.2	0.58
**F2**	7.2 ± 1.7	7.2 ± 1.5	0.46
	**RHH**	**LHH**	
**M1**	39.4 ± 2.3	39.3 ± 2.3	0.86
**M2**	39.8 ± 2.3	40.2 ± 2.2	0.63
**F1**	39.0 ± 1.6	39.4 ± 1.6	0.73
**F2**	39.2 ± 1.7	39.5 ± 1.8	0.68
	**TVH**	**MBH**	
**M1**	3.1 ± 0.6	40.4 ± 2.4	-
**M2**	3.3 ± 0.9	41.5 ± 2.2	-
**F1**	3.7 ± 0.3	40.1 ± 1.2	-
**F2**	3.6 ± 0.8	40.7 ± 1.8	-

All values are represented as averages ± standard deviations (mm). P-value refers to Student's t-test used to compare the averages between antimeres. RVH, height of the right ventricle; LVH, height of the left ventricle; RVW, width of the right ventricle; LVW, width of the left ventricle; RHH, height of the right hemisphere; LHH, height of the left hemisphere; TVH, height of the third ventricle; MBH, height of the brain at the midline.

On average, the ventricle measurements in older dogs (groups M2 and F2) were higher than those in younger dogs (groups M1 and F1). However, only LVH and LVW were significantly higher in older males (M2) than in younger males (M1) (p = 0.02 and p = 0.01, respectively) (Table 4).

**Table 4:** P-values of Student's t-test comparing the average of measurements taken between the four groups

**Measurements**	**M1 x M2**	**F1 x F2**	**M1 x F1**	**M2 x F2**
RVH	0.11	0.98	0.14	0.83
RVW	0.20	0.26	0.79	0.67
LVH	0.02*	0.43	0.47	0.96
LVW	0.01*	0.70	0.27	0.60
TVH	0.56	0.71	0.06	0.42
RHH	0.46	0.80	0.99	0.38
LHH	0.41	0.92	0.98	0.25

* p<0.05, significant difference.

RVH, height of the right ventricle; RVW, width of the right ventricle; LVH, height of the left ventricle; LVW, width of the left ventricle; TVH, height of the third ventricle; RHH, height of the right hemisphere; LHH, height of the left hemisphere.

**Figure 2:**
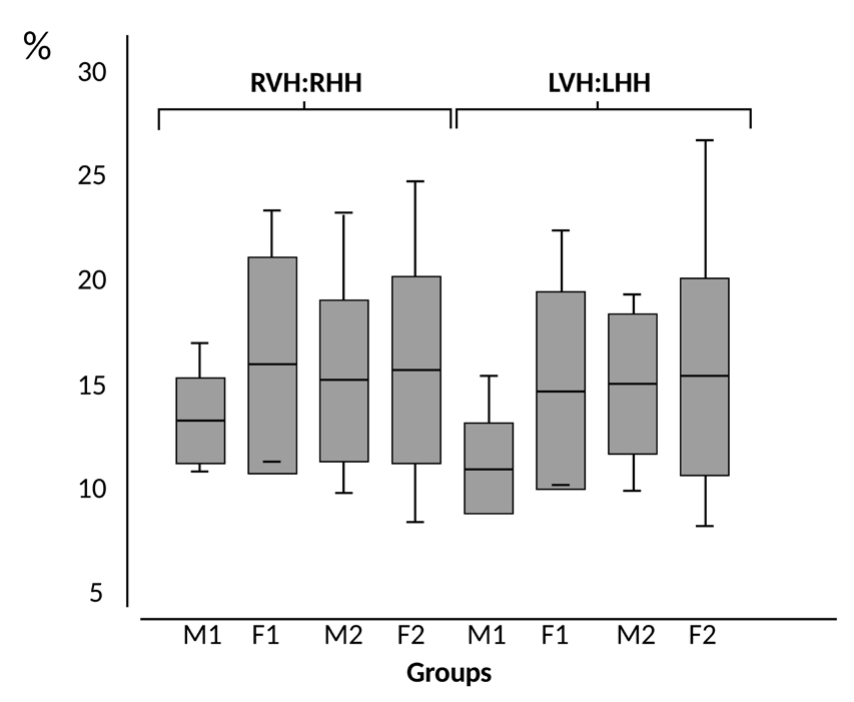
The boxplot shows the average and standard deviation of the RVH:RHH and LVH:LHH percentage ratios separated by age and sex groups (M1, F1, M2, F2). RVH, height of the right ventricle; RHH, height of the right hemisphere; LVH, height of the left ventricle; LHH, height of the left hemisphere.

In the whole sample (n = 55), the average RVH: RHH ratio was 15.3 ± 4.1%, LVH: LHH was 14.8 ± 4.3%, and TVH: MBH was 8.4 ± 2.1%. These ratios did not differ according to age and sex (Figure 2 and Table 5).

**Table 5:** Measurement ratios expressed in percentages

**Groups**	**Average** ± **Standard deviation (%)**		
	**RVH:RHH**	**LVH:LHH**	**TVH:MBH**
**M1**	13.3 ± 2.0	12.2 ± 4.0	7.8 ± 1.7
**M2**	15.3 ± 3.0	15.1 ± 3.4	7.8 ± 2.5
**F1**	16.0 ± 5.2	14.8 ± 4.7	9.1 ± 0.9
**F2**	15.8 ± 4.5	15.5 ± 4.8	8.8 ± 2.1
***p*-value**	0.46	0.26	0.30

All values are represented as averages ± standard deviations (%). P-values refer to a one-way ANOVA test to compare the ratios between groups. RVH, height of the right ventricle; RHH, height of the right hemisphere; LVH, height of the left ventricle; LHH, height of the left hemisphere; TVH, height of the third ventricle; MBH, height of the brain at the midline.

The ventricular system was clearly identified at the level of the interthalamic adhesions in all cases. Visualization of the septum pellucidum was possible in all animals and none of the images showed fused lateral ventricles. Of the 55 animals evaluated, only eight (14.5%) exhibited slight asymmetry between the lateral ventricles; five had a larger right lateral ventricle; and three demonstrated a larger left lateral ventricle.

## Discussion

On average, Poodles older than 11 years had slightly larger ventricles than younger dogs. Particularly, LVH and LVW were significantly higher (p < 0.05) in older than in younger males. At first glance, older dogs seem to have decreased cerebral tissue and, consequently, ventricles become proportionally larger with age, as previously described in a study on German Shepherd dogs (González-Soriano et al., 2001). However, the ratios between ventricles and cerebral heights did not support this inference in Poodle dogs, because there were no significant differences between these ratios in terms of sex, antimeres, or age. Similarly, other authors have calculated the ratios between ventricular and cerebral heights to interpret the ventricular sizes of different breeds and skull morphotypes (Doiche et al., 2022; Esteve-Ratsch et al., 2001; Kii et al., 1997; Vite et al., 1997; Woo et al., 2010).

A positive correlation among ventricular height, area, and volume has been previously reported in dogs (Woo et al., 2010). Thus, the values presented here (especially the ratios) can assist in interpreting ventricle sizes in Poodle dogs older than 7 years. However, because the transition zone between the dural extension and bone represents an area and not a specific landmark, slight differences in the measurements between distinct evaluators must be considered.

Doiche et al. (2022) conducted linear measurements of ventricular height using CT scans in 45 healthy dogs aged 2–5 years. In that study, the averages of RVH, LVH, and TVH were 3.8, 4.2, and 2.3 mm in German Shepherd; 3.9, 4.3, and 2.6 mm in Rottweiller; and 8.3, 8.3, and 2.6 mm in Boxer dogs, respectively. The present study identified that the mean RVH, LVH, and TVH sizes were 6.0, 5.8, and 3.4 mm for Poodle dogs, respectively. The RVH: RHH ratios were 11.35 mm, 9.56 mm, and 20.75%, and LVH:LHH ratios were 12.30 mm, 12.10 mm, 20.75% for German Shepherd, Rottweiller, and Boxer dogs, respectively (Doiche et al., 2022). In contrast, the RVH: RHH and LVH: LHH ratios in Poodles (15.3 and 14.8, respectively) were higher than those in German Shepherds and Rottweilers, but smaller than those in Boxer dogs.

Kii et al. (1997) examined 21 clinically normal Beagle dogs aged 1–2 years and identified that the average RVH and LVH were 4.7 and 4.6 mm, respectively. Since the four breeds analyzed by Doiche et al. (2022) and Kii et al. (1997) were larger than Poodles, it may be hypothesized that certain breed traits, rather than body mass or size, are more closely associated with differences in ventricle measurements in dogs. These results reinforce the necessity of establishing ventricular dimensions by breed, especially in those most commonly affected by neurological or behavioral problems.

Schröder et al. (2006) compared the measurement of ventricles in various breeds with different weights and skull conformations and reported that the differences were minor. Both Labradors and Poodles, who are mesaticephalic with varying body sizes, presented absolute linear measurements without statistical difference. Similar results were obtained when comparing dolichocephalic breeds (Dachshunds and German Shepherds). In another study, Pilegaard et al. (2017) inferred that the skull shape influences lateral ventricle volumes.

The RVH and LVH dimensions measured in ten clinically normal Yorkshire Terriers, aged 2–8 years, using MRI instead of CT-scan, were 4.06 ± 0.98 mm, and 4.47 ± 0.98 mm, respectively. The same study reported an average RVH: RHH of 15.6% and LVH: LHH of 17.1%. These values are very close to those obtained in Poodles (15.3% and 14.8%, respectively), possibly because they are both mesaticephalic breeds. Similarly, there were no significant differences in these ratios according to sex, age, and antimeres. In other mesaticephalic breeds, the average RVH: RHH was 9.6% and LVH: LHH was 12.1% in Labradors and Poodles (Schröder et al., 2006) and 12.2% and 12.1%, respectively, in Beagles (Kii et al., 1997).

Few studies have reported the width of the lateral ventricles in dogs. However, concurrent to the observations of Pilegaard et al. (2017), no significant differences were observed in the widths of the right and left lateral ventricles in this study.

An MRI study reported significant volumetric enlargement for both lateral ventricles in seven normal dogs rescanned after four years. These results suggested that ventricular enlargement during aging did not necessarily reflect observable pathological cognitive or behavioral changes (Gunde et al., 2020).

A slight to mild ventricular asymmetry is a common finding and may represent anatomical variations without clinical relevance in many cases. However, the frequency of asymmetry between the lateral ventricles in the Poodle dogs in our sample was low (14.6%) when compared with data from other studies (20% to 53.3%) (De Haan et al., 1994; Doiche et al., 2022; Kii et al., 1997; Pivetta et al., 2013; Schröder et al., 2006).

## Conclusion

Even though the averages of ventricular measurements tend to be higher in Poodles ≥11 years, the proportionality ratios indicate that this difference is insignificant. Therefore, ventricular measurements in senile Poodle dogs with symptoms of neurocognitive dysfunction may clarify whether these pathologies can change the size and ratios between the ventricles and brain in this breed.
